# Lithium causes differential effects on postsynaptic stability in normal and denervated neuromuscular synapses

**DOI:** 10.1038/s41598-021-96708-7

**Published:** 2021-08-26

**Authors:** Diego Zelada, Francisco J. Barrantes, Juan Pablo Henríquez

**Affiliations:** 1 Neuromuscular Studies Laboratory (NeSt Lab), Department of Cell Biology, CMA Bio-Bio, Facultad de Ciencias Biológicas, Universidad de Concepción, Casilla 160-C, Concepción, Chile; 2 Pontificia Universidad Católica Argentina (UCA)-Scientific and Technological Research Council of Argentina (CONICET), Buenos Aires, Argentina

**Keywords:** Neuromuscular junction, Bipolar disorder, Endocytosis

## Abstract

Lithium chloride has been widely used as a therapeutic mood stabilizer. Although cumulative evidence suggests that lithium plays modulatory effects on postsynaptic receptors, the underlying mechanism by which lithium regulates synaptic transmission has not been fully elucidated. In this work, by using the advantageous neuromuscular synapse, we evaluated the effect of lithium on the stability of postsynaptic nicotinic acetylcholine receptors (nAChRs) in vivo. We found that in normally innervated neuromuscular synapses, lithium chloride significantly decreased the turnover of nAChRs by reducing their internalization. A similar response was observed in CHO-K1/A5 cells expressing the adult muscle-type nAChRs. Strikingly, in denervated neuromuscular synapses, lithium led to enhanced nAChR turnover and density by increasing the incorporation of new nAChRs. Lithium also potentiated the formation of unstable nAChR clusters in non-synaptic regions of denervated muscle fibres. We found that denervation-dependent re-expression of the foetal nAChR γ-subunit was not altered by lithium. However, while denervation inhibits the distribution of β-catenin within endplates, lithium-treated fibres retain β-catenin staining in specific foci of the synaptic region. Collectively, our data reveal that lithium treatment differentially affects the stability of postsynaptic receptors in normal and denervated neuromuscular synapses in vivo, thus providing novel insights into the regulatory effects of lithium on synaptic organization and extending its potential therapeutic use in conditions affecting the peripheral nervous system.

## Introduction

Since the initial characterization of its therapeutic potential^1^, lithium has been widely used as a potent stabilizer agent for the treatment of bipolar disorder^2,3^. Although lithium treatment controls synaptic ‘over-excitation’ by down- and up-regulating the activity of excitatory and inhibitory synapses, respectively^4–6^, the involved downstream mechanisms are variable and likely depend on the cellular context^7^. For instance, in different synapses, lithium acts through inhibition of the glycogen synthase kinase-3β (GSK3β)^8,9^, up-regulation of neurotrophins and their receptors^10^, and reduction of the AMPA glutamate receptor levels in the cell surface^9,11–14^. In this regard, even though most of the molecular mechanisms described for lithium effects involve the activity^13^ and expression levels^15^ of postsynaptic receptors, the ability of lithium to regulate their stability has not been elucidated^13^.

The vertebrate neuromuscular junction (NMJ) is a peripheral cholinergic synapse formed by a presynaptic motor axon and the postsynaptic domain of a muscle fibre in a specialized region called the endplate. The NMJ has been broadly used to understand the principles of synaptic organization and function^16,17^. Along with its relatively large size and easier accessibility in comparison to central nervous system synapses, the availability of fluorescent conjugates of α-bungarotoxin (BTX), which binds with high affinity to muscle nicotinic acetylcholine receptors (nAChRs)^18^, have facilitated a deep understanding of postsynaptic organization at the NMJ, including the distribution, stability, and trafficking of nAChRs^19–24^.

In this work, we have evaluated the potential impact of lithium on the stability of muscle cell-surface nAChRs in individual NMJs in vivo*.* We found that lithium treatment exerts a differential effect on nAChRs turnover in normal and injured NMJs, attributable to decreased receptor internalization and increased incorporation at the muscle surface, respectively. In denervated muscle fibres, lithium potentiated the formation of unstable nAChR clusters in non-synaptic domains of the sarcolemma. Regarding the potentially involved mechanism, we found that lithium did not modify the denervation-dependent re-expression of the foetal nAChR γ-subunit. Denervation increased the total levels of the GSK3β target β-catenin in muscles but impairs its distribution within the endplate; however, lithium-treated denervated fibres retain β-catenin staining in specific foci of the synaptic region. Our findings reveal that lithium directly targets postsynaptic nAChR turnover at the NMJ and control their availability in synaptic and non-synaptic regions of normal and injured synapses.

## Results

### Compartmentalized analyses of nAChR turnover in mature NMJs

**Figure 1:**
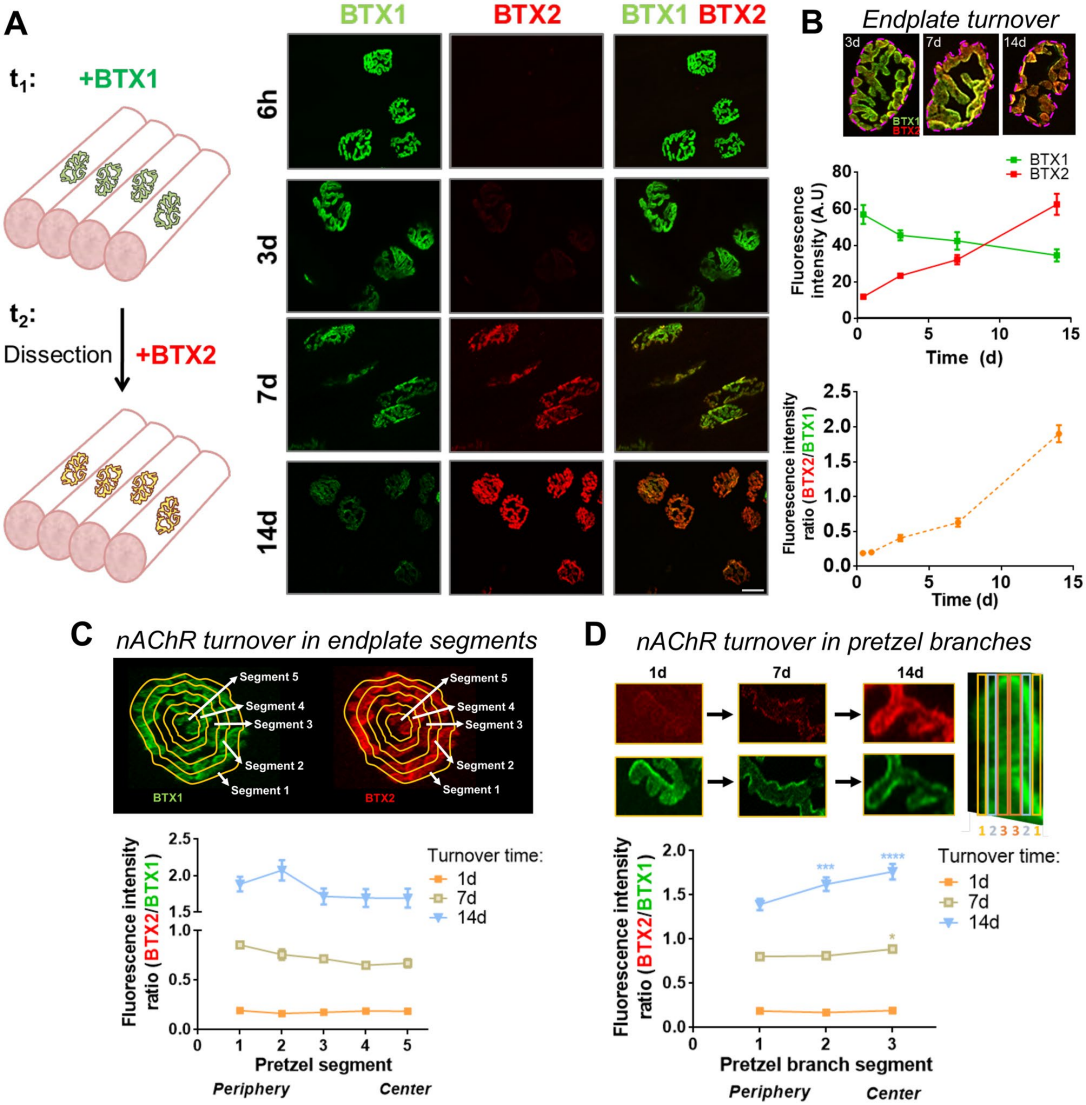
Compartmentalized analyses of nAChRs turnover in mature NMJs. (**A**) Experimental design used for the time-course analysis of nAChRs turnover. The pre-existing nAChRs pool was labelled with AlexaFluor488-BTX (BTX1) and after different time points, newly incorporated nAChRs were detected by AlexaFluor555-BTX (BTX2) staining. Pictures show representative confocal images of BTX1 and BTX2 staining at different time points ranging from 6h to 14d. Bar = 20 µm. (**B**) nAChRs turnover analyses in individual endplates were performed by quantifying BTX2 and BTX1 fluorescence intensity at different time points. nAChR turnover was evaluated using the BTX2/BTX1 fluorescence intensity ratio. Graphs represent mean values ± SEM of 25–50 NMJs for each time point. (**C**) Compartmentalized nAChRs turnover was determined by dividing each endplate in five concentric sections and fluorescence intensity of both BTX1 and BTX2 was measured. The graph represents mean values ± SEM for nAChRs turnover in each segment (1 to 5, from periphery to centre) from 10 endplates per animal. (**D**) nAChRs turnover within pretzel branches were analysed in three branches per pretzel located in the periphery, middle, and central region of the mature end-plate structure. Each branch was subsequently sectioned in peripheral (1, yellow), middle (2, gray), or centre (3, orange) segments. Fluorescence intensity ratios from BTX2/BTX1 were quantified and plotted. The graph represents the mean values ± SEM for each branch segment from 30 pretzel branches per animal. N = 2 (14d), or 3 (6h–7d). One-way ANOVA with subsequent Tukey’s multiple comparisons tests were performed at each point. ****p < 0.0001; ***p < 0.001; *p < 0.05 when middle (2) or centre (3) branch segments were compared to the peripheral (1) segment.

To determine the potential effects of lithium on the stability of postsynaptic nAChRs at the NMJ, we first studied the turnover of pre-existing by newly incorporated nAChRs on the surface of the postsynaptic muscle membrane by using a combination of fluorescently-conjugated BTXs. With this aim, pre-existing surface nAChRs were first labelled by an in vivo injection of AlexaFluor488-BTX on top of the cranial *Levator auris longus* (LAL) muscle (BTX1, Fig. 1A). After different time points, LAL muscles were dissected, fixed and newly incorporated nAChRs in the postsynapse were subsequently labelled with AlexaFluor555-BTX (BTX2, Fig. 1A). Confocal imaging using the same acquisition parameters revealed that initial BTX1 staining becomes gradually lost with time concomitantly with an increase in the newly incorporated nAChRs (BTX2) (Fig. 1A,B). Our observations also suggest that the incorporation of new nAChRs is not homogeneous in the plane of the endplate, as it becomes more evident towards the periphery at longer time intervals of nAChR turnover (Fig. 1B). To quantify this trend, we first subdivided individual endplates into five equivalent concentric areas and determined the BTX2/BTX1 intensity ratio in each of these regions. Quantification showed that at an early time point (1 day), an average BTX2/BTX1 ratio of 0.18 ± 0.10 was obtained throughout the pretzel structure (Fig. 1C). In turn, the turnover degree after 7 days was variable, with the uppermost value of ~ 0.9 in the periphery (segment 1), decreasing to ~ 0.7 at the centre of the endplate (segment 5). Furthermore, the turnover degree reached its maximum after 14 days, as the magnitudes of the BTX2/BTX1 ratio were ≥ 1.9 in the two outer segments (segments 1 and 2), and around 1.7 in the middle (segment 3) and more central regions (segments 4 and 5) of the endplate, respectively (Fig. 1C). In addition, we observed that nAChR turnover within individual branches of the postsynaptic structure was also progressively altered (Fig. 1D). Quantification of the relative BTX2/BTX1 intensity ratio in the periphery and the centre of each branch shows that whereas the BTX2/BTX1 ratio was around 0.2 in all branch segments after 1 day, a pronounced increase in newly incorporated nAChRs occurred at the centre of branches (segment 3), as the BTX2/BTX1 ratio reached values close to 0.9 (Fig. 1D). When surface nAChR turnover was measured in a time frame of 14 days, central branch segments displayed increased nAChR turnover, with BTX2/BTX1 ratio values of 1.62 ± 0.51 (segment 2) and 1.76 ± 0.59 (segment 3), respectively. Therefore, our analyses show that progressive nAChR turnover at increasing time intervals occurs in a compartmentalized fashion, as new nAChRs become incorporated in a differential pattern within the plane of the endplate as well as in individual pretzel branches.

### Lithium decreases nAChR turnover in mature NMJs

**Figure 2:**
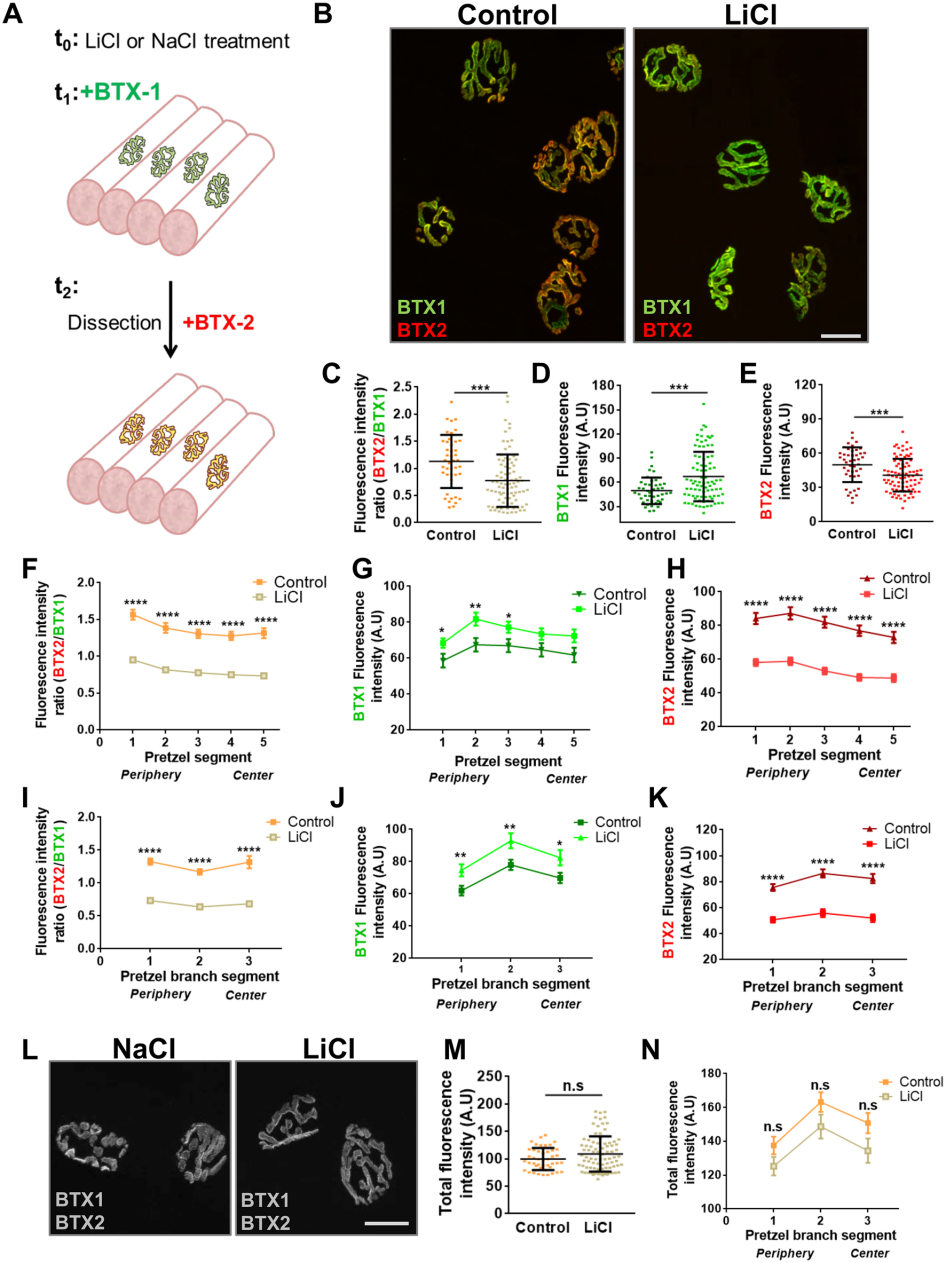
Lithium impairs nAChR turnover in normally innervated mature NMJs. (**A**) Experimental design used to analyse the effect of lithium on nAChRs turnover. Daily injection of control or lithium-treated groups was performed 3 days before BTX1 labelling. After 7 days, BTX2 was used to stain newly incorporated nAChRs. Confocal imaging of BTX1 or BTX2 within individual mature nAChR structures is represented for control (NaCl) or lithium (LiCl)-treated mice. (**B**) Pictures show representative confocal images of merged BTX1 and BTX2 staining after lithium or control treatment. Bar = 20 µm. (**C**) nAChRs turnover was determined by quantifying the BTX2/BTX1 fluorescence intensity ratio. BTX1 (**D**) and BTX2 (**E**) fluorescence intensities are shown to determine the contribution of each nAChR pool in all tested conditions. Graphs represent means ± SD along with individual data for control or lithium condition. For statistical analyses, unpaired *t*-test was used. ***p < 0.001; **p < 0.01. Detailed analyses of lithium effect on nAChRs turnover in segmented endplates (**F–H**) or segmented pretzel branches (**I–K**) were performed by quantifying the BTX2/BTX1 fluorescence intensity ratio for each experimental group. Multiple t-tests were carried out for statistical analysis between each segment from control or lithium-treated groups respectively. ****p < 0.0001; **p < 0.01; *p < 0.05. (**L**) Confocal imaging of BTX1 plus BTX2 fluorescence signals are displayed in gray colour to facilitate comparison on nAChRs density between experimental conditions. The total fluorescence signal of BTX1 plus BTX2 was measured both for endplate (**M**) (Bar = 20 µm) and postsynaptic branches (**N**). Graphs in (**M**) represent mean values ± SD along with individual data for control or lithium condition. *t*-test was performed for statistical analyses of 30–40 NMJs per animal (N = 3 animals per group). n.s: not significant; p > 0.05.

Based on our initial findings showing that nAChRs turnover progresses almost linearly from 6 h to 14 days (Fig. 1B,D), we reasoned that potential variations of the process caused by lithium could be efficiently detected and quantified by comparing BTX2/BTX1 ratios at 7 days. In these experiments, daily subcutaneous injections of LiCl or control NaCl were applied on top of LAL muscles from three days previous to BTX1 staining and until BTX2 application (Fig. 2A). Confocal imaging showed that lithium-treated muscles exhibit an evident decrease in the amount of BTX2 staining (Fig. 2B). Quantification shows that the BTX2/BTX1 intensity ratio was significantly impaired in the lithium-treated group (0.979 ± 0.046), as compared to controls (1.280 ± 0.069; *p* < 0.001, t-test) (Fig. 2C). Further quantification showed that the impairment in nAChR turnover induced by lithium was attributable to a reduced removal of pre-existing nAChRs (Fig. 2D) and also to a significantly decreased incorporation of new nAChRs (Fig. 2E). Lithium treatment did not alter nAChR distribution within the postsynaptic domain, as the patterns of fluorescence intensity within the endplate (Fig. 2F–H) or in individual pretzel branches (Fig. 2I–K) were similar to controls. Indeed, quantification of nAChR turnover revealed that lithium treatment reduced the BTX2/BTX1 ratio in all measured segments of postsynaptic structures (Fig. 2F,I). Importantly, the decreased nAChR turnover induced by lithium was due to persistent staining of the pre-existing nAChR pool (Fig. 2G,J) accompanied by impaired incorporation of new nAChRs either in the entire endplate or in individual pretzel branches (Fig. 2H,K). Finally, to evaluate the possibility that lithium treatment increased the density of nAChRs in the postsynaptic domain, we quantified BTX1 plus BTX2 fluorescence intensities in individual pretzels (Fig. 2L–N). We found no substantial differences in total BTX staining between control and lithium-treated groups at the endplate (Fig. 2M) or in individual segments of postsynaptic branches (Fig. 2N). Together, these results reveal that lithium treatment exerts an inhibitory effect on nAChR turnover in normally innervated neuromuscular synapses.

### The NMJ structure is not significantly altered by lithium treatment

**Figure 3:**
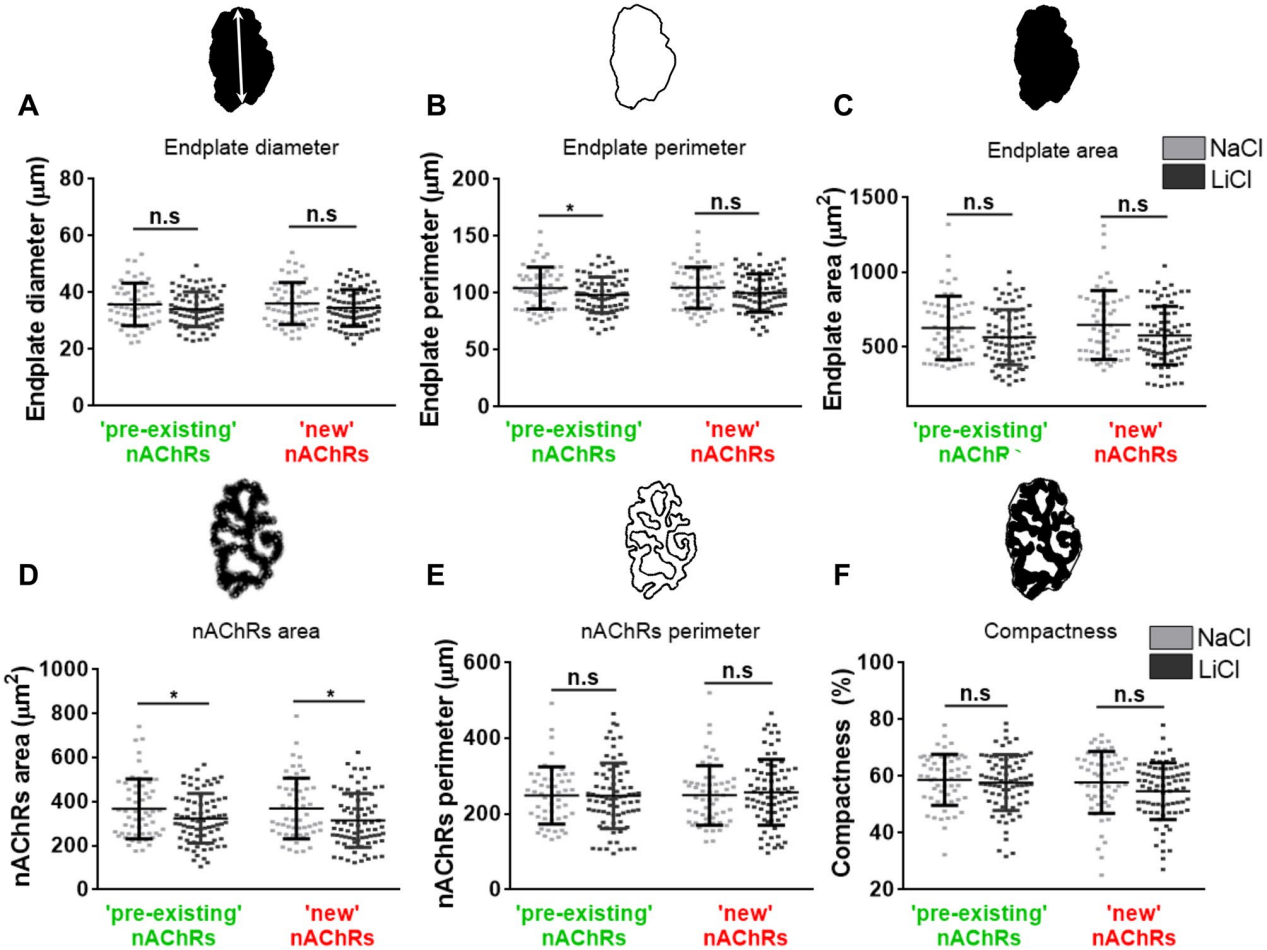
Postsynaptic NMJ morphology is not affected by lithium treatment. LAL muscles from control- or lithium-treated groups were subjected to the two-colour BTX assay. Images from BTX1 and BTX2 staining from lithium- and control-treated muscle samples were subjected to a systematic approach to perform morphometric analyses at the NMJ to determine postsynaptic parameters including endplate diameter (**A**), perimeter (**B**), and area (**C**). We also determined nAChRs morphological parameters, such as nAChR area (**D**), perimeter (**E**), and compactness (**F**). Graphs represent the mean values ± SD along with individual data from 30 to 40 NMJs per animal (N = 3 animals per group). *t*-test were performed for pre-existing (BTX1) or new (BTX2) nAChRs, respectively. *p < 0.05.

nAChRs turnover is severely compromised in pathological conditions associated with major changes in postsynaptic morphology, such as NMJ denervation and muscle diseases^25–28^. To evaluate whether the effect of lithium on nAChR turnover also affected the organization of mature NMJs we next performed systematic morphometric analyses of postsynaptic parameters (Fig. 3). Our findings showed that nAChR distribution at the endplate was not severely affected by lithium treatment, as endplate diameter and area in lithium-treated samples did not differ from controls (Fig. 3A–C). This was consistent with nAChR cluster analyses, as no significant effect of lithium was observed on the nAChR perimeter. Even though the area of both pre-existing and new nAChRs slightly decreases (Fig. 3D), the relative area occupied by nAChR clusters within the endplate (i.e. ‘compactness’) was not significantly different in both groups (Fig. 3D–F). To analyse in detail the potential effect of lithium on NMJ presynaptic organization, motor axon active zones and pre-existing nAChRs were stained with anti-piccolo and 488Alexa-BTX (BTX1), respectively, and NMJs were analysed by Structured Illumination Microscopy (SIM). As previously reported, piccolo distributes in a punctate pattern intercalated between nAChRs-enriched stripes within the postsynaptic domain (Supplementary Figure S1)^29,30^. The NMJs of both NaCl- and LiCl-treated mice display comparable densities and distribution of piccolo puncta, revealing that lithium treatment does not significantly alter presynaptic organization (Supplementary Figure S1). Based on our findings showing that the comparatively high levels of pre-existing nAChRs induced by lithium were not related to alterations in the density or distribution of nAChRs in mature postsynaptic apparatuses (Figs. 2, 3), we aimed our next analyses to nAChR trafficking.

### Lithium inhibits nAChR internalization in CHO-K1/A5 cells

**Figure 4:**
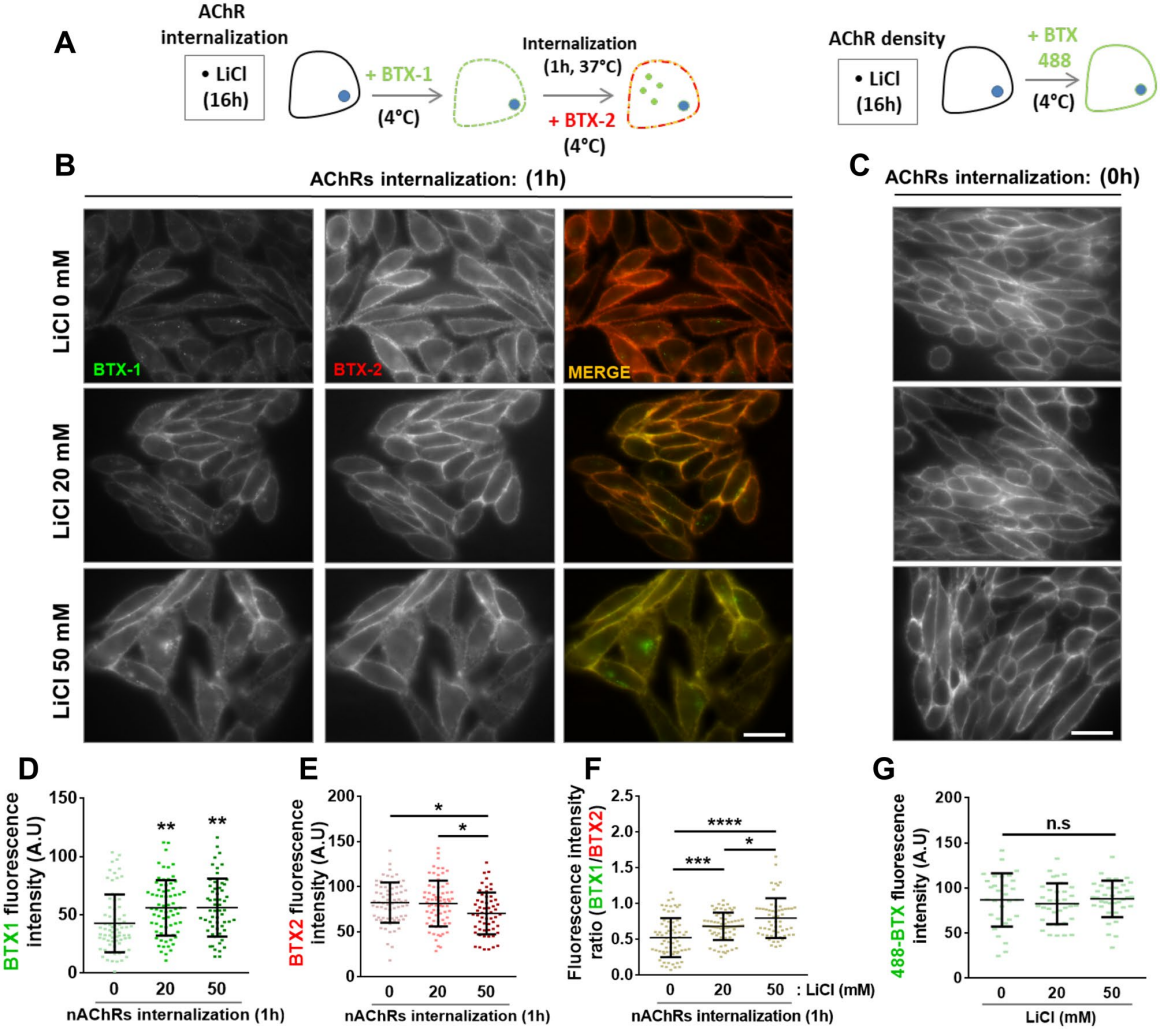
Lithium negatively regulates nAChR turnover in CHO-K1/A5 cells. (**A**) Experimental design used to analyse the effect of lithium on nAChR internalization in CHO-K1/A5. Briefly, cells were incubated overnight with PBS or LiCl at 20 and 50 mM. Then, AlexaFluor555-BTX (BTX1, pseudo-coloured green) was used to label surface nAChRs at 4 °C. After receptors were allowed to internalize for 1 h at 37 °C, AlexaFluor488-BTX (BTX2, pseudo-coloured red) was used to label new nAChRs at the cell surface. To evaluate the effect of lithium on nAChRs density, control- or lithium-treated cells were incubated with AlexaFluor488-BTX for 45 min at 4 °C and then fixed for subsequent fluorescence microscopy visualization. (**B**) Fluorescence microscopy imaging of CHO-K1/A5 cells labelled with BTX1 and BTX2 revealed the degree of nAChR turnover at the cell surface after 1 h of internalization. (**C**) Fluorescence microscopy imaging of CHO-K1/A5 cells labelled only with AlexaFluor488-BTX after treatment with LiCl or PBS was performed to determine nAChRs density at the cell surface. Bar = 50 µm. Mean fluorescence intensity for (**D**) BTX1 or (**E**) BTX2 at the cell surface after 1 h of nAChR internalization was quantified. Also, (**F**) BTX1/BTX2 fluorescence intensity ratio at the cell surface was measured and represented in respective graphs as means ± SD along with individual values of two independent experiments. For each group, the membrane-associated fluorescence intensity of 60–80 CHO-K1/A5 cells was analysed. One-way ANOVA and Tukey’s multiple comparisons test were performed for each condition. ****p < 0.0001; ***p < 0.001; **p < 0.01; *p < 0.05.

As a first hint to evaluate if lithium treatment affects nAChR internalization we used heterologous CHO-K1/A5 cells expressing the muscle-type nAChRs, as these cells have facilitated the identification of several factors controlling nAChR trafficking and stability at the cell surface^31–35^. In these experiments, cells were incubated without or with 20 or 50 mM LiCl for 16 h. Then, surface nAChRs were first labelled with AlexaFluor555-BTX (BTX1, pseudo-coloured green) and after 1 h of internalization newly incorporated nAChRs were labelled with AlexaFluor488-BTX (BTX2, pseudo-coloured red) (Fig. 4A, left panel). Fluorescence imaging revealed that increments in lithium concentration (20 and 50 mM) persistently led to relatively higher BTX1 staining at the cell surface (Fig. 4B, middle and bottom panels), suggesting that lithium treatment inhibits nAChR internalization. Quantification showed that the initially labelled nAChRs remained at the cell surface for a longer period, as revealed by the increase in BTX1 fluorescence intensity in lithium-treated cells (Fig. 4D), whereas the incorporation of new nAChRs (BTX2) was inhibited in these cells (Fig. 4E), which led to increased BTX1/BTX2 ratio at the cell surface, concomitant to the increment of lithium concentration (Fig. 4F). These results reveal that lithium impairs the removal of nAChRs from the cell surface, which leads to reduced nAChR turnover. Next, to distinguish whether the higher levels of BTX1 staining are attributable to impaired internalization or to increased initial nAChR levels at the cell surface, control- and lithium-treated cells were incubated with AlexaFluor488-BTX alone (Fig. 4A, right panel). Our findings show that fluorescence intensities were similar amongst all experimental groups (Fig. 4C,G). These findings reveal that rather than affecting total nAChR density at the cell surface, lithium inhibits nAChR internalization.

### Lithium enhances nAChR turnover in denervated NMJs

**Figure 5:**
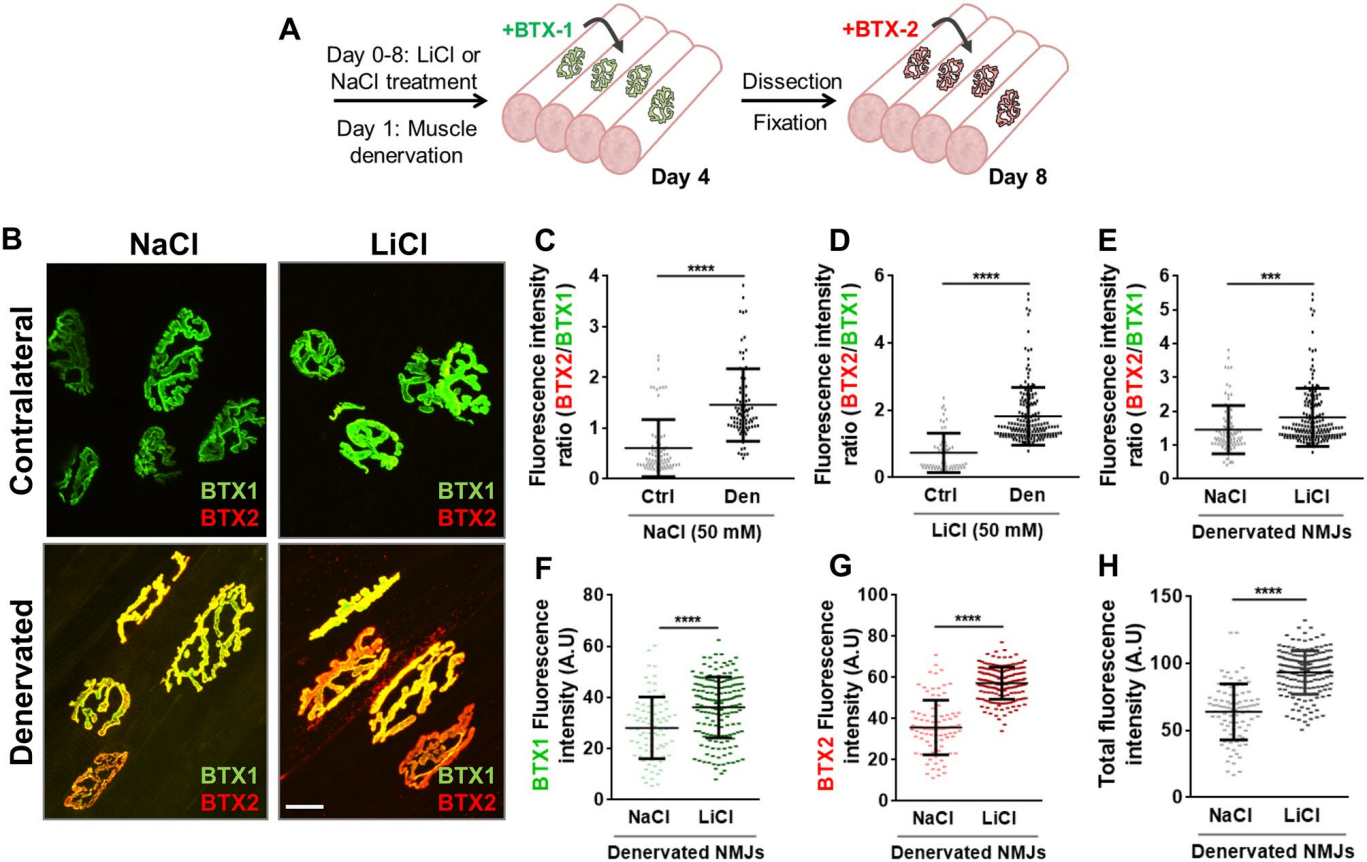
Lithium stimulates nAChR turnover and density in denervated NMJs. (**A**) Experimental design used to analyse the effect of lithium on nAChRs turnover in denervated NMJs. Since one day before facial nerve cut, lithium or sodium chloride were subcutaneously injected on top of the LAL muscle daily until BTX2 labelling. The initial surface nAChR pool was labelled 3 days after facial nerve cut (BTX1) while newly incorporated nAChRs (BTX2) were stained 7 days after nerve cut. (**B**) Pictures show representative confocal images of merged BTX1 and BTX2 staining in denervated and contralateral LAL muscles from sodium or lithium-treated animals. Bar = 20 µm. (**C–E**) nAChRs turnover was determined by quantifying the BTX2/BTX1 fluorescence intensity ratio. BTX1 (**F**) and BTX2 (**G**) fluorescence intensities are shown to determine the contribution of each nAChR pool in all tested conditions. (**H**) Total fluorescence signal of BTX1 plus BTX2 in entire endplates was also quantified. Graphs represent mean values ± SD along with individual data for sodium or lithium condition. For statistical analyses, a *t*-test was employed for comparison between controls and lithium-treated groups from 30 to 40 NMJs per animal (N = 3 animals per group). ****p < 0.0001; ***p < 0.001; **p < 0.01.

Previous findings have shown that lithium reduces the number of nAChRs in the absence of motor neuron input and in primary muscle cell cultures^36,37^ but the potential mechanisms involved are unknown. Therefore, we next explored the effect of lithium treatment on postsynaptic stability after LAL muscle denervation inflicted by a 4 mm resection of the facial nerve^38^ (Fig. 5A). As an increase in nAChRs turnover is expected after denervation^39^, in these experiments we shortened the time frame between BTX1 and BTX2 incubations to 4 days to increase the detection range obtained with the same acquisition parameters used in our previous experiments. As expected, denervation induced a marked increase in nAChR turnover, evidenced by higher BTX2 than BTX1 staining in NaCl- or LiCl-treated muscles compared to their contralateral muscles (Fig. 5B–D). However, in contrast to what was observed in uninjured NMJs (Fig. 2), lithium treatment in denervated mice also resulted in more pronounced incorporation of new nAChRs (Fig. 5B); indeed, quantification shows that lithium induces a significant increase in nAChR turnover compared to the denervated NaCl-treated group (Fig. 5E). As in innervated NMJs, lithium treatment also resulted in an increased pool of pre-existing nAChRs (compare Fig. 5F with Figs. 2D,G,J). However, opposite to uninjured NMJs, the levels of newly incorporated nAChRs at the muscle surface were significantly increased by lithium treatment (Fig. 5G). Consequently, higher nAChR density was observed at the surface of lithium-treated muscles as compared to the NaCl-treated group (Fig. 5H). Altogether, these data reveal that upon NMJ denervation lithium enhances the availability of nAChRs by increasing the levels of both pre-existing and newly incorporated receptors within the NMJ postsynaptic domain.

### Lithium induces the aggregation of unstable nAChR clusters in denervated NMJs

**Figure 6:**
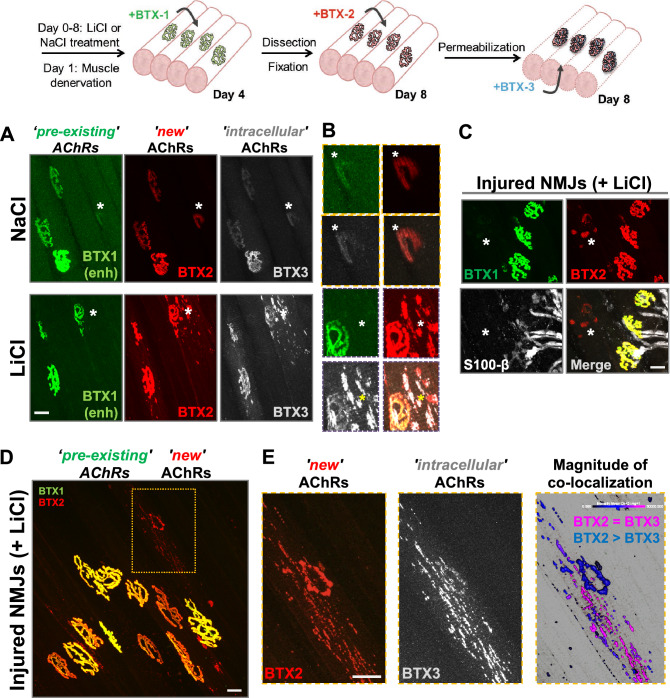
Lithium stimulates the formation of unstable nAChR clusters in non-synaptic regions of denervated muscle fibres. (**A**) Experimental design used to address the origin and stability of denervation-associated nAChR clusters. Since one day before facial nerve cut, lithium or sodium (control) chloride were subcutaneously injected on top of the LAL muscle daily until BTX2 labelling. The initial surface nAChR pool was labelled 3 days after facial nerve cut (BTX1) while newly incorporated nAChRs (BTX2) were stained 7 days after nerve cut. Intracellular nAChRs were labelled by tissue permeabilization and subsequent incubation with AlexaFluor647-BTX (BTX3, ‘intracellular’ nAChRs). Confocal imaging was performed along entire muscle fibres both in synaptic and non-synaptic regions of the sarcolemma. The AlexaFluor488-BTX fluorescence intensity was digitally enhanced (BTX1 enh) to evidence positive or negative BTX1 staining in non-synaptic regions (asterisks) for each experimental condition. Bar = 20 µm. (**B**) Representative higher magnification images of non-synaptic regions for each condition. Bar = 20 µm. (**C**) To analyse if BTX2-only structures detected in lithium-treated denervated muscles were located in non-synaptic muscle regions, immunohistochemistry for Schwann cells using anti S100-β was performed along with BTX1 and BTX2 staining on mice subjected to facial nerve cut. Bar = 20 µm. (**D**) Further characterization of non-synaptic nAChR clusters is evidenced by co-localization of BTX2 and BTX3. (**E**) Higher magnification images of the dotted line square in D reveal total or partial co-localization between newly incorporated nAChRs (BTX2) and intracellular nAChRs (BTX3) after IMARIS® analyses. Briefly, regions where BTX2 signal was also positive for BTX3 are pseudo-coloured magenta (BTX2 = BTX3). In turn, regions positive for BTX2 with undetectable BTX3 signal are pseudo-coloured blue (BTX2 > BTX3). N = 3 animals per group. Bar = 20 µm.

An early hallmark of NMJ denervation is the assembly of ectopic nAChR aggregates in the muscle membrane^40–42^. As in hind-limb muscles, we found that LAL muscle denervation induced nAChR *aggregates* exhibiting either plaque- or oval-like shapes, which displayed an average volume of 39.92 ± 6.37 µm^3^ and strong BTX2 staining (Fig. 6A,B, asterisks). To confirm that nAChR aggregates were distributed in non-synaptic regions of the sarcolemma, we took advantage of the fact that previously innervated NMJs maintain Schwann cells staining for some days after denervation^43^. Therefore, we performed immunofluorescence detection of myelin-forming and perisynaptic terminal Schwann cells along with BTX1 and BTX2 labelling (Fig. 6C). We consistently found that perisynaptic Schwann cells contacted exclusively denervated pretzel-like structures having both BTX1 and BTX2 staining; in turn, nAChR aggregates not contacted by Schwann cells were smaller and almost exclusively positive for BTX2 labelling (Fig. 6C). Remarkably, we found that lithium treatment specifically induced the aggregation of small nAChR *clusters* (volume ≤ 5 µm^3^, Supplementary Figure S2) distributed within the vicinity of denervation-dependent ectopic aggregates (Fig. 6A,B,D). Even though both ectopic nAChR aggregates and clusters share preferential BTX2 staining, evident remnants of BTX1 staining were only visible in control (NaCl-denervated) conditions, revealing that lithium destabilizes non-synaptic nAChRs (Fig. 6B). To discriminate if ectopic nAChR structures result from reduced stability (i.e. BTX1 staining reduction) or increased incorporation of new nAChR aggregates (i.e. higher BTX2 staining) from intracellular compartments, we included a third labelling step of intracellular nAChRs with AlexaFluor647-BTX (BTX3) after tissue permeabilization (Fig. 6A,B,E). Our results showed that intracellular nAChRs displayed a markedly preferential co-distribution with ectopic clusters rather than with ectopic nAChR aggregates, suggesting that lithium-induced nAChR clusters arise mainly from the contribution of an intracellular pool of nAChRs (Fig. 6E). Altogether, our studies demonstrate that lithium exerts differential effects on the stability of the postsynaptic domain in both innervated and denervated NMJs.

### Lithium alters the distribution of β-catenin within the endplate

**Figure 7:**
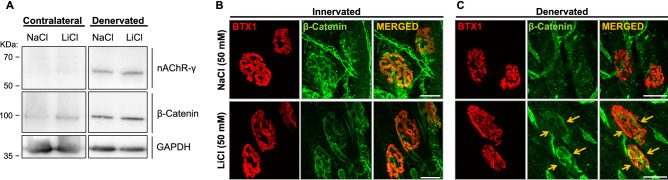
Lithium restricts the localization of β-catenin and nAChR γ-subunit at the NMJ. (**A**) Total protein samples of LAL muscles from NaCl- and LiCl-treated mice were analysed by Western blot using specific antibodies to detect the nAChR γ-subunit (upper panel) or β-catenin (middle panel). The levels of GAPDH were used as loading control (bottom panel). (**B**,**C**) Whole-mounted LAL muscles were subjected to immunofluorescence analyses to detect β-catenin (pseudocoloured green) in uninjured (**B**) and denervated (**C**) NMJs treated with 50 mM NaCl or LiCl. Pre-existing nAChRs were labeled in vivo with 488Alexa-BTX (BTX1, pseudocoloured red). Arrows show β-catenin staining in specific foci within the endplate of lithium-treated fibres. Bar = 20 µm.

We next analysed the levels and distribution of specific proteins potentially involved in the effects of lithium on NMJ stability. First, we considered evidence showing that denervated NMJs re-express the foetal nAChR γ-subunit, which is normally replaced by the adult ε-subunit during postnatal development^42,44^. Considering that foetal nAChR pentamers display faster turnover than adult nAChRs^45,46^, we analysed the potential effect of lithium on the expression of the nAChR γ-subunit. As expected, innervated muscle extracts contained almost undetectable levels of the nAChR γ-subunit (Fig. 7A). Although denervation induced a strong increase in a ~ 60 kDa immunopositive band, the abundance of the nAChR γ-subunit was not significantly altered by lithium treatment (Fig. 7A). Secondly, we considered that amongst its downstream effectors, lithium inhibits GSK3β, which leads to the cytosolic accumulation of β-catenin, a central mediator of both canonical Wnt signalling activity and cell–cell interactions^8,9^. Interestingly, lithium treatment rescues NMJ alterations in mice expressing a truncated form of the muscle specific kinase receptor (MuSK), a central mediator of nAChR aggregation, likely via increasing β-catenin distribution in the endplate area^47^. Therefore, we studied the potential involvement of β-catenin on the lithium-mediated modulation of nAChR stability. Western blot analyses showed that lithium treatment of control innervated mice results in the accumulation of a ~ 100 kDa β-catenin immunoreactive band compared to sodium-treated mice (Fig. 7A). LAL muscle denervation triggers a strong β-catenin increase in control NaCl-treated mice, which was not further altered by lithium treatment (Fig. 7A). Immunofluorescence analyses showed that β-catenin distributed in both presynaptic axons and within the endplate in control innervated muscles (Fig. 7B), a pattern that was not significantly modified by lithium treatment. Interestingly, even though muscle denervation strongly decreased β-catenin detection in the endplate region of NaCl-treated mice, lithium-treated fibres retain β-catenin staining in specific foci of the synaptic region (Fig. 7C). Therefore, while muscle denervation increases the levels of β-catenin and the nAChR γ-subunit, lithium does not modify the levels of these proteins; however, the distribution of β-catenin is locally modified by lithium.

## Discussion

To analyse the potential effects of lithium on surface nAChR stability, we have set up in vivo assays in the NMJs of the cranial LAL muscle, a flat, thin, and superficially exposed muscle that facilitates repeated in vivo visualization and manipulation^38,48–51^. In addition, we have optimized efficient NMJ denervation in LAL muscles^38^. Compared to other labelling strategies, the use of BTX conjugated to different fluorophores at different times in individual NMJs has proven to be particularly advantageous as it allows to (1) separately discern changes in both pre-existing and newly-incorporated nAChR pools, (2) analyse the overall and compartmentalized distribution of nAChRs, and (3) estimate postsynaptic receptor density^19,27^. We found that a time frame of 7 days between a first AChRs staining with Alexafluor488-BTX (named as ‘BTX1’) and a second staining with Alexafluor555-BTX (named as ‘BTX2’) allows efficient quantification of nAChR turnover within the same endplate in vivo, consistent with previous findings in hind-limb muscles showing that surface nAChRs exhibit half-life times of around 14 days^19,20,52^. We also found preferential incorporation of nAChRs towards the periphery of endplates, similar to previous findings obtained in denervated muscles^53^ and in aneural laminin-dependent complex postsynaptic structures formed in vitro^54^.

One first main observation of our current studies in non-injured NMJs is that lithium exposure led to a marked decrease in nAChRs turnover in the absence of alterations in receptor density. Although previous reports showed that lithium reduced the number of nAChRs in skeletal muscle^36,55^, some important differences in the experimental design could potentially account for these discrepancies, such as the relatively higher lithium dose (intraperitoneal 400 mM twice daily), the use of [^125^I]-BTX for nAChRs labelling and quantification^36^, and that the analyses were performed in the all-slow twitch Soleus muscle, which could have some still unknown nAChR turnover differences with the mainly fast-twitch LAL muscle used in our present studies. Despite these differences, further experiments performed by the same authors in cultured myotubes showed that the insertion rate of new nAChRs on the cell surface was significantly reduced by lithium^55^, as we observed in innervated NMJs.

What are the mechanisms involved in the effect of lithium on nAChR stability at the cell membrane? Cumulative evidence obtained in heterologous CHO-K1/A5 cells expressing the mature form of muscle nAChRs has allowed the identification of several factors and mechanisms controlling muscle-type nAChR internalization and stability at the cell surface^32–35^. From these studies, it has been proposed that nAChRs endocytosis follows a two-step process: first, receptors are removed and sequestered within the proximity of the plasma membrane, and second, nAChRs traffic *en route* through early/late endosomes where they are degraded by a lysosome-dependent mechanism^32^. While this mechanism requires actin polymerization and Rac1 GTPase activity, it is independent of the classic clathrin, caveolin, or dynamin pathways^32^. In cultured myotubes, initial studies suggested that lithium entered muscle cells through cation channels and affected nAChR trafficking in a similar fashion to innervation and calcium, likely via an inositol-dependent pathway^55^. Interestingly, in vitro experiments where nAChR aggregation on the cell surface of myotubes was stimulated with the neural-derived protein agrin showed that lithium promotes nAChRs dispersion through a relatively late mechanism occurring after the required tyrosine phosphorylation of its β-subunit, likely dependent on GSK-3β inhibition, with the subsequent disruption of a cytoskeleton scaffolding^56^. Consistent with our current observations in uninjured NMJs, lithium had no effect on nAChRs density at the surface of CHO-K1/A5 cells but inhibited receptor internalization and turnover degree, which opens the possibility that lithium regulates crucial effectors of nAChRs internalization. In this context, lithium treatment prevents Rac1 localization in the lamellipodia of keratinocytes^57^ and decreases the GTPase activity of Rac1 in primary monocytes^58^. As a consequence, lithium-dependent impairment of Rac1 function disorganizes actin distribution and inhibits lamellipodia formation and cell migration^57,58^. Thus, one possibility is that lithium inhibits nAChRs internalization through local signalling that alters a Rac1-dependent actin scaffolding required for proper receptor endocytosis. An additional interesting possibility involves the presynaptic machinery, as electrophysiological studies using the crayfish NMJ showed that the replacement of sodium ions by lithium in the external solution resulted in decreased transmitter release and inhibited excitability of presynaptic terminals^59^, a mechanism that evokes nAChRs instability resulting from pre- or postsynaptic activity blockade^19^. In this regard, our resolutive SIM analyses showed that lithium did not cause significant alterations in the organization of presynaptic active zones.

A second main observation of our present studies is that lithium further increased nAChRs instability and turnover occurring in denervated NMJs. Remarkably, this response was due to inhibition of nAChR internalization accompanied by enhanced incorporation of ‘new’ nAChRs, which collectively led to the establishment of postsynaptic domains containing a relatively higher density of nAChRs. The idea that lithium impacts muscle postsynaptic machinery by enhancing the availability of nAChRs is also supported by the observation that most intracellular nAChRs were already incorporated at the NMJ at the time of initial nAChR staining in lithium-pretreated denervated muscles. Denervated muscles re-express the foetal nAChR γ-subunit, as expected^42,44,60^; however, lithium treatment did not significantly modify its expression. In this regard, although lithium-treated denervated NMJs share reduced nAChRs stability with foetal NMJs, the latter also display impaired conductance and calcium permeability^61–63^. These findings suggest that lithium effects at the NMJ do not involve the nAChR γ-subunit and support the idea that lithium increases the availability and density of nAChRs to improve synaptic transmission upon reinnervation. Consistently, lithium promoted the formation of nAChR clusters in non-synaptic regions, which co-localization with intracellular nAChRs suggest that they are *en route* towards the muscle surface.

Patients suffering bipolar disorders exhibit increased excitatory glutamatergic and dopaminergic transmission and reduced inhibitory GABAergic activity^64–66^. Even though these functional synaptic imbalances have been efficiently counteracted by lithium treatment, the potential mechanism involved remains unclear. Whereas lithium treatment has been shown to regulate the levels and activity of postsynaptic receptors^13,15^, its impact on receptor stability is unknown. Collectively, our data reveal that lithium differentially regulates the stability of nAChRs in normal and denervated neuromuscular synapses in vivo, providing new insights into the mechanism governing the therapeutic effects of lithium for its use in neuropsychiatric disorders. In addition, even though lithium has also been used to treat different types of dystrophies, as it enhances skeletal muscle strength^67,68^, the potential involvement of the neuromuscular synapse in these therapeutic responses has not been fully elucidated. Relevant evidence has been obtained in mutant mice expressing a truncated form of MuSK, a crucial receptor for nAChR aggregation and function at the NMJ ^47,69^. Mice expressing a truncated form of MuSK that lacks the cysteine-rich ligand-binding domain of MuSK (CRD) displayed aberrant postsynaptic organization and function that correlated with β-catenin exclusion from the endplate region^47^. Interestingly, lithium treatment rescued NMJ alterations and β-catenin localization within the synaptic region^47^. In line with this evidence, we found that the NMJs of lithium-treated denervated muscles preserved β-catenin staining within the endplate region. Similar to previous studies^47^, lithium-dependent redistribution of β-catenin did not resemble the pretzel-like shape of the mature NMJ that we observed in innervated controls but localized in specific foci at the endplate. This observation opens the possibility that lithium exerts an increment of β-catenin levels in other resident cell types of the NMJ niche that become active upon denervation^70–72^. Altogether, considering the high prevalence of neuromuscular diseases having the NMJ as a primary target, our studies also reveal the potential use of lithium as a complementary treatment to target nAChRs stability in pathologies associated with the muscular or motor systems.

## Material and methods

### Animals and in vivo treatments

CF-1 and Swiss-Webster mice were maintained at 20–26 °C, with dark/light cycles of 12 h and fed with pellet (Prolab RMH-3000, LabDiet) and water ad libitum*.* All carried experimental procedures were approved by the Bioethics Committee at Universidad de Concepción, Chile, and followed the norms imposed by the Bioethics Committee of the National Research and Development Agency, Chile (ANID). All animal procedures complied with ARRIVE^73^ and were conducted in adult mice (3–4 months old) under sedation (2.5% v/v isofluorane with a 0.8–1 L/min oxygen mixture). Anesthetized mice were subjected to a daily single subcutaneous injection of 50 mM LiCl (Sigma-Aldrich) on the top of LAL muscles diluted in sterile PBS at room temperature. Depending of the experimental approach, mice were treated for consecutive 8 or 10 days. Control-treated groups were similarly injected with a 50 mM NaCl (Merck Millipore) solution. Before LAL muscle dissection, animals were euthanized by an overdose of isofluorane.

### Facial nerve injury

Facial nerve resection was performed as described^38^. Briefly, mice were maintained anesthetized with a mixture of 2.5% v/v isoflurane and 0.8–1 L/min oxygen and a surgical 5 mm skin incision was performed to expose facial nerve branches. The posterior auricular branch of the facial nerve (which innervates the LAL muscle) was cleared and a 4 mm section of the facial nerve branch was carefully transected. Then, the skin was sutured using absorbable monofilament surgical suture (Ethicon Vicryl USP 6–0) and animals were periodically monitored until their recovery. Contralateral LAL muscles were used as a control uninjured condition in LiCl- or NaCl-treated mice.

### Two color α-bungarotoxin assay

To analyse nAChRs dynamic at the NMJ in vivo, pre-existing nAChRs at the muscle surface were labelled on anesthetized mice with a non-saturating dose (4 μg/mL) of AlexaFluor488-conjugated BTX (BTX1; Molecular Probes) injected subcutaneously on the top of LAL muscles. After different time points (6 h; 3, 7, or 14 days), mice were sacrificed and the LAL muscles were quickly dissected, washed, and fixed with 0.5% v/v formaldehyde (Merck-Millipore) for 1.5 h at room temperature. Then, LAL samples were washed with ice-cold PBS and newly incorporated nAChRs were labelled with AlexaFluor555-conjugated BTX for 1 h (BTX2, diluted 2 μg/mL in PBS) at room temperature. In some experiments, after BTX1 and BTX2 staining, muscles were permeabilized with PBS containing 0.5% v/v Triton X-100 for 2 h at room temperature and incubated overnight at 4 °C with AlexaFluor647-conjugated BTX (BTX3, diluted 2 μg/mL in PBS) to detect intracellular nAChR subpopulations. Samples were washed with PBS and mounted for confocal microscopy visualization. Images (*z*-stacks) were captured with 40X (Plan-Apochromat 40x/1.3 Oil DIC M27) and collected at 1 μm intervals in a Zeiss LSM 700 confocal microscope at the CMA Bio-Bio facility (University of Concepcion, Chile). All acquisition parameters were equally maintained between control- and lithium conditions. To determine nAChRs abundance on each condition, pretzel-like structures were manually delineated by using ‘free-hand selections’ on Image J software version 1.53e (https://imagej.nih.gov/ij/), and BTX1 or BTX2 mean fluorescence intensity were measured. BTX1 or BTX2 values were subsequently used for nAChRs turnover analyses. For NMJ morphometric analyses, endplate perimeter, area, or diameter as well as nAChRs area, perimeter, and compactness were performed according to the ‘NMJ-morph’ systematic analyses^74^. To reveal total or partial co-localization between newly incorporated nAChRs (BTX2) and intracellular nAChRs (BTX3), z-stack images were processed with the IMARIS® software version 9.2 (https://imaris.oxinst.com).

### Compartmentalized analyses of nAChRs distribution within endplates

To analyse nAChRs distribution within the endplate plane, postsynaptic pretzel-like structures were manually delineated and five identical concentric segments were automatically obtained using Image J. On each of these segments, regions lacking nAChRs were discarded by setting a threshold of 10.0 arbitrary units of fluorescence intensity. Thus, only BTX1 or BTX2 positive staining were included for mean fluorescence intensity recording. nAChRs distribution within pretzel branches was evaluated by enclosing a rectangle on branches located either at the periphery or centre of the postsynaptic structures, where three pairs of rectangular segments were created from the outermost to the innermost part of each branch. Then, the mean fluorescence intensity of BTX1 or BTX2 was measured in each segment and nAChRs turnover was calculated as the BTX2/BTX1 fluorescence intensity ratio.

### NMJ immunostaining

After BTX1 and BTX2 staining, whole-mounted LAL muscles were incubated with 0.15 M glycine for 30 min. After several washing steps with 0.01 M PBS/0.5% v/v Triton X-100 for 2 h, samples were blocked with 4% w/v BSA dissolved in PBS/0.5% Triton X-100. Primary antibodies against S100-β (1:300, DAKO), β-catenin (1:1000, Millipore), or piccolo (1:300; Synaptic Systems #142 003) were incubated overnight in blocking solution at 4 °C. After washing, samples were incubated with the secondary antibodies (Cy3 or Cy5 1:250; Donkey H + L, Jackson Immunoresearch Laboratories) overnight at 4 °C, and subsequently mounted between two coverslips in DAKO fluorescence medium. Confocal z-plane optical sections were acquired as mentioned above. In some experiments, mounted samples were visualized by Structural Illumination Microscopy (Zeiss ELYRA S1- SIM super-resolution laser microscope) and images were captured with 63X (Plan-Apochromat 63x/1.4 Oil DIC M27) objective.

### Western blot

Dissected LAL muscles were homogenized in RIPA buffer (150 mM NaCl, 1 mM EDTA, 1% NP-40, 0.5% sodium deoxycholate, 0.1% SDS, 50 mM Tris pH 8.0) containing a cocktail of protease inhibitors (Halt™ Protease Inhibitor Cocktail 100X; #78430, Thermo ScientificTM) along with 2 mM Na_3_VO_4_. For immunoblotting, proteins were separated in 10% sodium dodecyl sulphate–polyacrylamide gel electrophoresis (SDS-PAGE), transferred onto polyvinylidene difluoride (PVDF) membranes (Thermo Scientific), and probed with rabbit anti β-catenin (1:1000, Millipore), rabbit anti nAChR γ-subunit (1:1000, Invitrogen), or mouse anti GAPDH (1:1000; Santa Cruz Biotechnologies). Bound antibodies were visualized using the respective HRP-coupled secondary antibodies (Jackson ImmunoResearch Laboratories) developed using a chemiluminescence reagent kit (Perkin Elmer).

### nAChRs internalization assays in CHO-K1/A5 cells

CHO-K1/A5 cells were generated by transfection of the Chinese hamster ovary (CHO) cell line with the genes corresponding to the four adult mouse nAChR subunits^31^. Cells were cultured in growing media consisting in Ham’s F-12 medium (Thermo Fisher) supplemented with 10% v/v FBS at 37 °C with 5% CO_2_. For lithium treatment, a solution of 20 or 50 mM of LiCl (or PBS alone as control) was prepared in growing media and cells were incubated for 16 h at 37 °C. nAChRs located at the cell surface were labelled with AlexaFluor555-BTX (BTX1, 4 μg/ml) diluted in PBS containing 0.6 mM CaCl_2_ and 1.6 mM MgCl_2_ (PBS-Ca^2+^/Mg^2+^) for 45 min on ice. Thereafter, cells were washed with PBS-Ca^2+^/Mg^2+^ to remove any excess of BTX1 and nAChRs were allowed to internalize for 1 h in growing media at 37 °C. Next, newly incorporated nAChRs were labelled with AlexaFluor488-BTX (BTX2, 4 μg/ml) in PBS-Ca^2+^/Mg^2+^ for 45 min on ice and cells were fixed in 2% paraformaldehyde for 20 min at 4 °C, as described^32^. After 2 washes with ice-cold PBS, cells were mounted and visualized by conventional fluorescence microscopy. nAChRs internalization and turnover was evaluated measuring the relative fluorescence intensity of BTX1 and BTX2 at the cell surface. To evaluate nAChRs density, control or LiCl-treated cells were incubated with AlexaFluor488-BTX (4 μg/ml) in PBS-Ca^2+^/Mg^2+^ for 45 min on ice, and cells were fixed and mounted as described above.

### Statistical analyses

All data were analysed using GraphPad Prism software version 6.0 for Windows (www.graphpad.com). All experiments were performed three independent times by triplicate, unless it is indicated. To analyse the effects of NaCl or LiCl on nAChRs turnover and density in non-injured or denervated NMJs, 20–50 synapses from each animal were measured and compared between groups. For studies of nAChR turnover and density in CHOK1/A5 cells, a total of 60–80 cells per group were compared from randomly acquired fluorescence images. Statistical analyses were performed using the Student’s t-test of unpaired data for lithium- and control-treated comparison. Comparisons involving three variables were performed with one-way ANOVA followed by Tukey’s post-hoc test. A *p* value of < 0.05 indicated statistically significant differences.

## Supplementary Information

Supplementary Figures.
