# Supplementary material for: Lithium causes differential effects on postsynaptic stability in normal and denervated neuromuscular synapses
Source: Sci Rep. 2021 Aug 26;11:17285. doi: 10.1038/s41598-021-96708-7 (PMC8390761; doi:10.1038/s41598-021-96708-7)
Supplement: Supplementary file 1 — Supplementary Figures. [file 41598_2021_96708_MOESM1_ESM.pdf]

Supplementary information

Lithium causes differential effects on postsynaptic stability in normal and denervated neuromuscular synapses

Diego Zelada[1], Francisco J. Barrantes[2] and Juan Pablo Henríquez[1*]

[1]Neuromuscular Studies Laboratory (NeSt Lab), Department of Cell Biology, CMA Bio-Bio, Universidad de Concepción, Chile.

[2]Pontificia Universidad Católica Argentina (UCA)-Scientific and Technological Research Council of Argentina (CONICET), Buenos Aires, Argentina.

[*] Correspondence should be addressed to:

Juan Pablo Henríquez. Departamento de Biología Celular, Facultad de Ciencias Biológicas, Universidad de Concepción, Concepción, Chile. Casilla 160-C, Concepción, Chile. Phone: 56-41-2203492; FAX: 56-41-2245975; EMAIL: jhenriquez@udec.cl

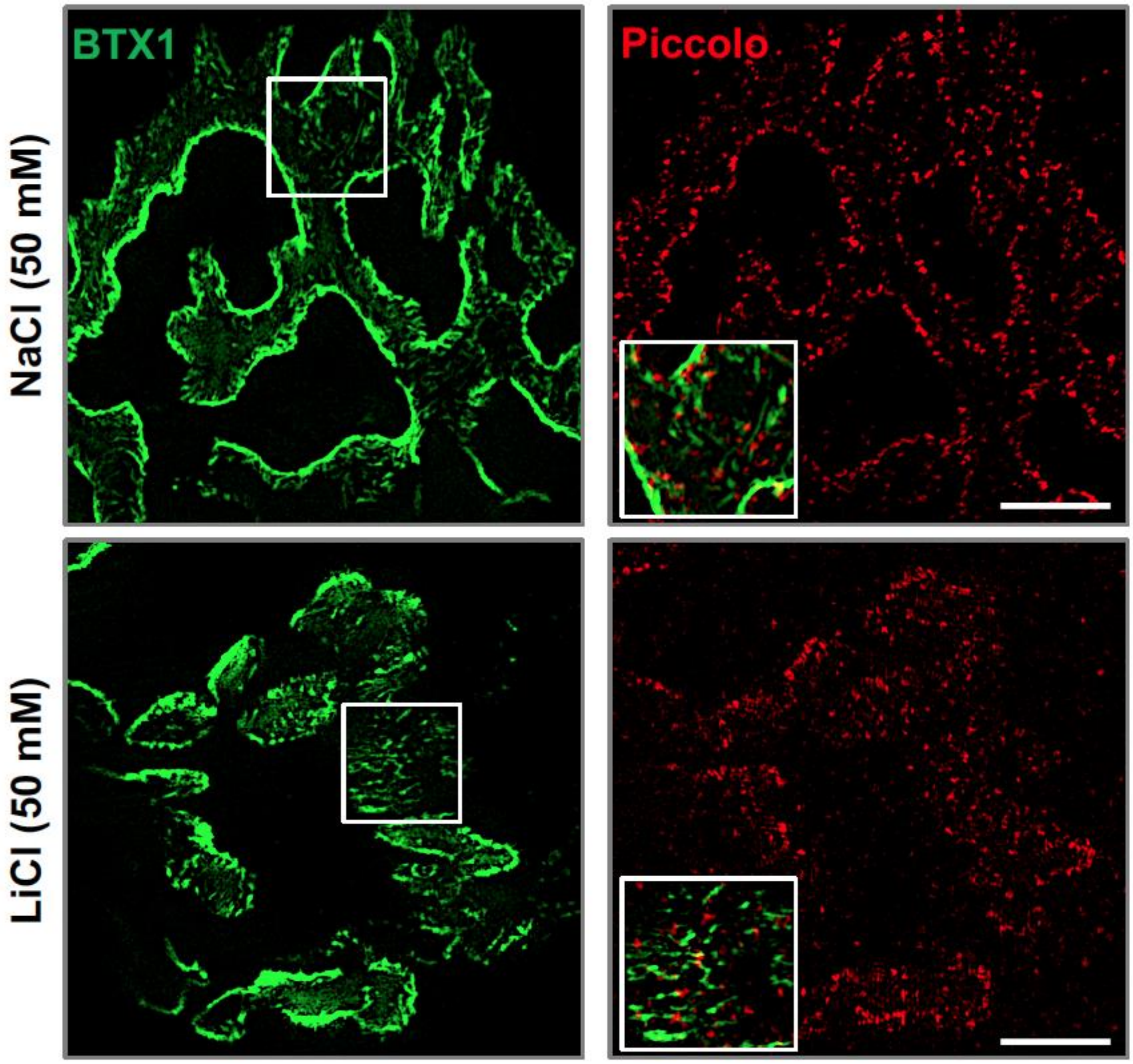

Supplementary Figure S1. Lithium does not significantly alter the NMJ pre and postsynaptic organization.

Pre-existing nAChR aggregates at the NMJs of LAL muscles from NaCl- or LiCl-treated mice were stained with 488Alexa-BTX (BTX1, green) *in vivo.* After 7 days, muscles were dissected, fixed, and processed for immunofluorescence with anti-piccolo (red). Pre- and postsynaptic apposition at NMJs was analysed by Structured Illumination Microscopy. Insets show merged magnified images of the white line square region in each condition. Bar = 5 µm.

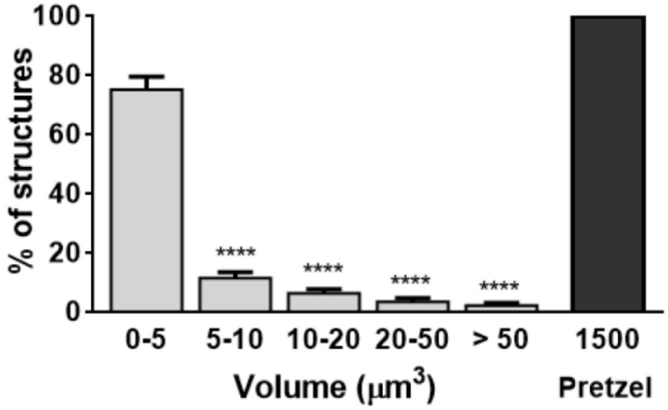

Supplementary Figure S2. Analyses of nAChR cluster volume.

Data were represented as means ± SEM of the percentage of structures exhibiting volumes of 0-5, 5-10, 10-20, 20-50, and > 50 μm^3 respectively. Moreover, the pretzel volume was measured for comparison with non-synaptic nAChR structures using the IMARIS® software version 9.2 (https://imaris.oxinst.com). One-way ANOVA and Tukey's multiple comparisons test were performed for each condition. ****$p < 0.0001$ when each range of volume was compared with the 0-5 μm^3 range.

A

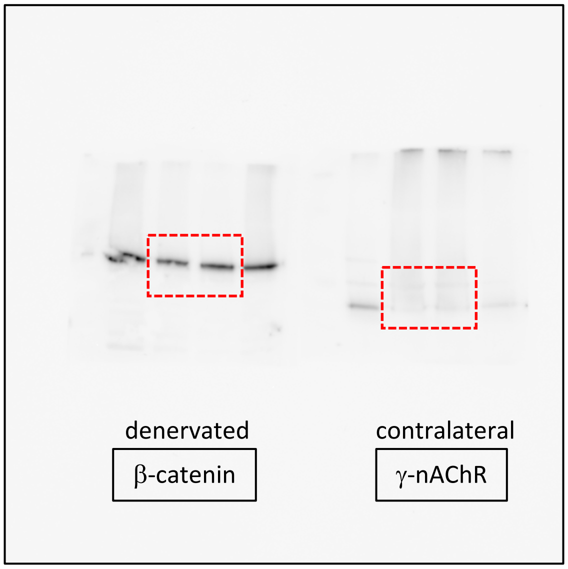
denervated
β-catenin
contralateral
γ-nAChR

B

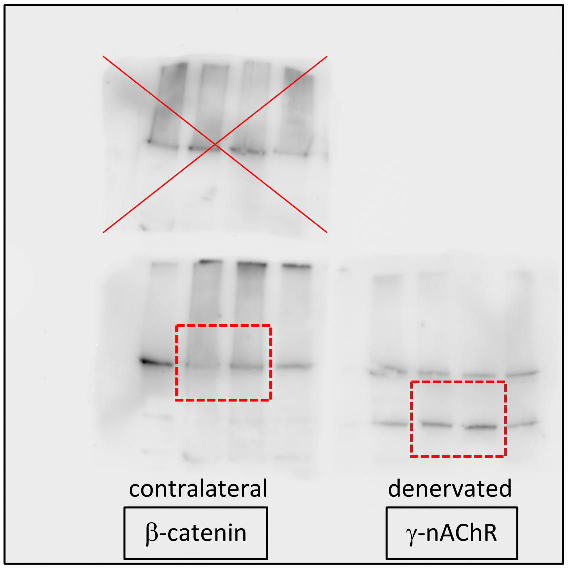
contralateral
β-catenin
denervated
γ-nAChR

C

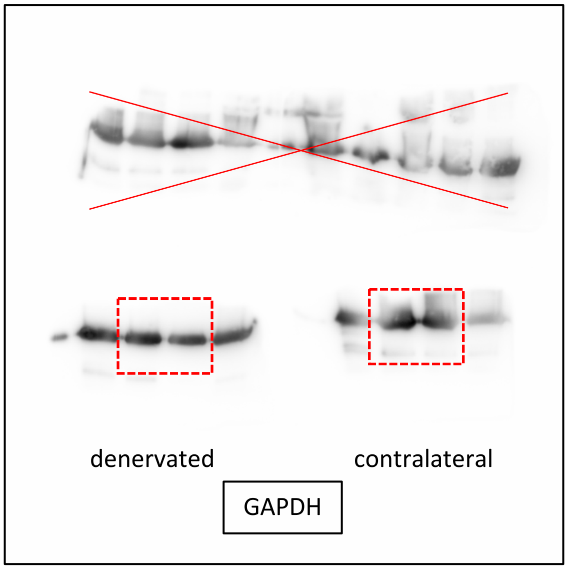
denervated
contralateral
GAPDH

Supplementary Figure S3. Effect of lithium on the expression levels of NMJ proteins – blot images.

Total protein samples of denervated and contralateral (control) LAL muscles from mice treated with NaCl (lanes 1 and 2) or LiCl (lanes 3 and 4) were analysed by Western blot using specific antibodies to detect β-catenin (A,B) or the nAChR γ-subunit (A,B). The levels of GAPDH (C) were used as loading control.
